# Climate Change Warning Labels on Gas Pumps: The Role of Public Opinion Formation in Climate Change Mitigation Policies

**DOI:** 10.1002/gch2.202000086

**Published:** 2021-07-16

**Authors:** James R. Brooks, Kristie L. Ebi

**Affiliations:** ^1^ Think Beyond the Pump P.O. Box 1675 Kapaau HI 96755 USA; ^2^ University of Washington 4225 Roosevelt Way NE #100 Seattle WA 98105 USA

**Keywords:** carbon labels, climate change philosophy, social norms marketing, transportation emissions policies

## Abstract

This article analyzes City of Cambridge, Massachusetts legislation that requires all gasoline and diesel pumps to display a consumer warning label outlining the climate change and public health impacts from fuel combustion. This review of empirical and theoretical scholarship on efficacy of carbon label programs and health warning labels suggests government‐sponsored “warming labels” may increase self‐efficacy beliefs. The analysis reveals warming labels may activate extant climate concern norms and shift public opinion toward long term support of sustainable transportation emissions policies and practices.

## Graphical abstract

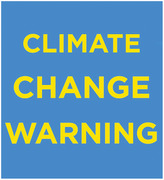

## 1 Introduction

Low carbon transport is a critical element of climate policies. Where participation and commitment of citizens can pave the way for its immediate and long‐term successful implementation.^[^ ^1^ ^]^ Product labels warning of the latent harms from combustion of petroleum‐based transportation fuels (2020 updated cost of health impacts on air quality along with climate damages from combustion is estimated at $6.50 per gallon of regular, non‐diesel gasoline)^[^ ^2^ ^]^ are an immediate emotional nudge to consumers^[^ ^3^ ^]^ that may facilitate the transition to a low‐carbon future.

Nascent government initiatives in Sweden,^[^ ^4^ ^]^ Cambridge, MA,^[^ ^5^ ^]^ and North Vancouver, BC^[^ ^6^ ^]^ place a consumer climate change‐public health warning label or “warming labels” on all transportation fuel pumps. These labels indicate that continuing to burn fossil fuels is a moral liability from human actions. Although labels may not radically shift consumer behavior, they can improve an audiences’ ability to make choices and alter preferences.^[^ ^7^ ^]^ This, in turn could promote climate policies and actions to reverse unacceptable practices creating harms for human health and well‐being.^[^ ^1^ ^]^

Warming labels schemes can be initiated by a local government or private agency. A public standard carbon label is where government sets the standards and monitors the program, while a private voluntary standard is owned and operated by a private company.^[^ ^8^ ^]^ Private voluntary labels can generate emissions reduction through choosing lower carbon options in purchasing decisions.^[^ ^9^ ^]^ Sweden is an example of a public standard carbon label on a gas pump promoting lower carbon options. Sweden will use a graded scale of greenhouse gas intensity, that is, labels display a “climate impact” rating system for conventional fuels, biofuel, and EV charging stations, along with the renewable share and raw materials of the fuel. Sweden's eco‐labels utilize preference for lower carbon fuels (and energy sources) designed to nudge consumers toward less carbon‐intense energy sources. North Vancouver, BC is an example of a “hybrid” public standard carbon label initiated by government but controlled and disseminated by the Canadian Fuels Association. Ambiguously promoting private interest, this “Smart Fuelling” program is an example of oil interest intentionally decorating themselves as environmental protectors—which can distort the market for carbon label programs.^[^ ^8^ ^]^

We review scholarship on how exposure to a public health warning label on a gas pump, focusing on the City of Cambridge, Massachusetts measure may shift climate change perceptions, beliefs, attitudes, behaviors, and policy support. The Cambridge public standard scheme will place a bright yellow, text‐only warming label on the front‐center‐face of a gas pump, stating “Burning Gasoline, Diesel, and Ethanol has major consequences on human health and on the environment including contributing to climate change.” The message by city government is an attempt to regulate petroleum‐based transportation fuels as a public health and environmental menace. The message reflects constitutional constraints or government “compelled speech” of a private company.

We highlight the crucial role of domain‐specific communication about anthropogenic climate change in influencing individual and group attitudes toward mitigation.^[^ ^1^ ^]^ Government‐sponsored warming labels as trusted, experiential education about combustion harms may effectively address the climate knowledge‐action gap. By activating extant climate concern norms (e.g., social norms), Cambridge warming labels engage the public as crucial actors in enacting and adopting low carbon transport.

We particularly highlight the relationships between social norms marketing in altering preferences, increasing self‐efficacy beliefs, and shaping the public opinion of autonomous individuals. Based on these findings, we propose directions for future research and best practices for researchers seeking to understand the relationship between warming labels, social norms, and climate action.

## 2 Results

### 2.1 Labels are Likely Positive Communication Mechanisms for Climate Action

Labels can act as “guilt” appeals or can be designed to correct misperceptions to motivate climate action.

As a “guilt appeal,” the Cambridge text may generate a strong sense of responsibility, of surveillance, and a warning that punishment could occur that could become in a circular fashion, a norm itself.^[^ ^10^ ^]^ Positive framing in climate messaging however, stimulates action more than negative.^[^ ^1^ ^]^ At the same time, when someone perceives a (potentially) threatening message as significant and personally relevant, that person experiences strong negative emotional responses like fear and worry.^[^ ^11^ ^]^ Thus, there may be an optimal level of negativity where fear plays an important role in motivating discussions about climate change.^[^ ^1^ ^]^

Warming labels may increase self‐efficacy beliefs when the messaging feels personally relevant so that individuals feel uniquely called upon (e.g., responsible for combustion harms) to reduce the harms through their own actions.^[^ ^12^ ^]^ Self‐efficacy belief is a perceived ability to perform a recommended response, and the perceived effectiveness of the recommended response to avoid the threat determines whether the response to the threatening message is adaptive (e.g., changes attitudes, intentions, or behavior consistent with the message) or maladaptive (resistance or dismissal to/of the message).^[^ ^11^ ^]^ A primary measurement of warming label effectiveness may be the degree to which warming labels increase self‐efficacy beliefs.

Warming labels take into account that knowledge is a social process embedded in social practices, identities, norms, conventions, and institutions.^[^ ^13^ ^]^ As such, they are a social marketing scheme that may correct for pluralistic ignorance. Pluralistic ignorance is a tendency to misjudge what the beliefs and actions of others actually are. Americans currently underestimate the political consensus on climate change (Ballew et al. study found 69% of Americans agree that global warming is happening. Whereas Leiserowitz found 55% agree).^[^ ^14^, ^15^ ^]^ On policy attitudes, majorities think government should do more to reduce the effects of climate change,^[^ ^16^ ^]^ including regulating carbon dioxide (CO_2_) as a pollutant.^[^ ^17^ ^]^ Basic environmental awareness and social responsibility motivation are key to a warming label's ability to motivate greenhouse gas reductions.^[^ ^8^ ^]^

Warming labels may legitimate everyday interpersonal discussions about climate change where pluralistic ignorance is a barrier to such discussions, which reduces effectively taking collective action.^[^ ^14^, ^18^ ^]^ Reminding the public that a majority of Americans share their concern may produce strong (e.g., positive) desires to adopt peer group expectations and increase peer‐group pressure in climate change mitigation.^[^ ^7^, ^19^, ^20^, ^21^, ^22^ ^]^ Warming labels may promote climate concern norms already present and inform others that a majority share their concern.^[^ ^18^ ^]^

### 2.2 Warming Labels may Establish Trust

The public interest, educational role of government warming labels about commonly misunderstood, negative effects from combustion may effectively establish trust^[^ ^23^ ^]^ and thus increase the likelihood of their acceptance and practice by decision‐makers.^[^ ^1^ ^]^ Considering the Cambridge text for example, research found the long residence time of CO_2_^[^ ^24^ ^]^ and negative public health consequences from combustion are poorly understood by most people^[^ ^21^ ^]^ (Gill et al. suggested label text incorporates this message; “Burning fossil fuels worsens the climate emergency with major projected health impacts increasing over time”).^[^ ^25^ ^]^ The Cambridge text could act like “present risk management tools” reducing uncertainty, complexity, and intangibility to a present here and now where the future must be taken into account.^[^ ^1^ ^]^

Further, warming labels may indicate how other members of a social group are expected to think and act in specific situations.^[^ ^20^ ^]^ Trust is a mediating factor in carbon label efficacy, facilitating evaluation of scientific claims about the environment, making information easier to understand that shape public opinion.^[^ ^26^ ^]^ Trusted warming labels connecting climate‐health harms to individual's routine consumption of fuel could build support for further emissions reductions in transportation.

### 2.3 Trusted Science is a Key to Self‐Efficacy

A trusted source of climate change information for most Americans regardless of political affiliation comes from climate scientists.^[^ ^27^ ^]^ Brewer et al. and Sampei et al. note trusted climate change information using appropriate timing and context could be powerful across the political spectrum.^[^ ^26^, ^28^ ^]^

Public interest warming labels utilizing trust in government science is a factor in building self‐efficacy beliefs. According to Rhodes et al. and Perkins et al., trust is significant in achieving implementation of effective emission mitigation policies.^[^ ^27^, ^29^ ^]^ Receiving climate change information through trusted sources would strengthen pro‐environmental stances socially and would give special authority in interpersonal communications; considered the most important in changing attitudes and beliefs.^[^ ^26^, ^29^, ^30^, ^31^ ^]^

### 2.4 Alarmed Appeals can Shape Public Opinion

Warming labels may be the only way for a minority (e.g., alarmed opinion) to gauge public opinion and to feel free to express their own in the social environment.^[^ ^32^ ^]^ They provide the social‐environmental pressure that creates public opinion mainly because individuals find that others think as they do, sanctioning their position.^[^ ^33^, ^34^ ^]^ Warming labels may provide a strong normative environment to facilitate the policy changes needed to address climate change^[^ ^35^ ^]^ by determining who can speak and who cannot, which plays a leading role in public opinion formation.^[^ ^33^ ^]^

Warming labels may legitimize and anchor minority opinion by lessening the fear alarmed individuals may have about speaking competently or appearing antagonistic when speaking to others about climate change and increasing interpersonal discussions and opinion expression about climate change.^[^ ^14^, ^18^, ^32^, ^33^, ^34^ ^]^

### 2.5 Warming Labels can Elevate the Social Status of Those Alarmed About Climate Change

An “alarmed” minority (26%) of Americans are fully engaged in and addressing the reality and seriousness of climate change personally and politically—in contrast to a political majority “concerned” but yet not engaged.^[^ ^15^ ^]^ The strength of this not‐alarmed majority in the social environment has the power to punish or sanction minority opinion.^[^ ^33^ ^]^

Media is crucial in focusing attention and constructing behavioral possibilities while limiting counter arguments or evidence.^[^ ^36^ ^]^ Warming labels could utilize the role media has as agent of the environmental socialization process.^[^ ^37^ ^]^ Media messages, such as warming labels that broadly increase the salience of climate urgency, could liberate its importance in dominant public opinion.^[^ ^33^ ^]^

### 2.6 Warming Labels Communicate Beyond Self‐Selected Audiences

Climate change information tends to reach already concerned audiences, with acceptance of climate science occurring along a liberal/conservative continuum that do not reach broader audiences (e.g., preaching to the choir).^[^ ^28^, ^38^, ^39^ ^]^ Alternative climate science communication efforts would not depend on individuals seeking climate change information out of an already held concern but would occur consistently in their everyday lives.^[^ ^28^, ^35^, ^38^ ^]^

Trusted warming label's point of sale (e.g., domain‐specific) location would reach all petroleum fuel consumers. Domain‐specific communication signaling a collective action happening now would end the practice of traditional climate change communication tending to address individuals as isolated actors while not changing the meaning and materials associated with social practices as a more effective way to increase self‐efficacy beliefs.^[^ ^38^ ^]^ Further, “psyche‐wise” messages about climate change and health can counteract a tendency for individuals to think their individual efforts are insignificant and won't exert an effort unless others do.^[^ ^40^ ^]^

Cambridge labels at the pump use an experiential learning approach to climate change communication, emphasizing where learning happens through firsthand, rather than others’ experience.^[^ ^41^ ^]^ Such messaging can facilitate understanding of the cause and effect of man‐made climate change as perpetrated by and negatively affecting us, here and now.^[^ ^27^, ^28^ ^]^

Public standard carbon labels are more trusted than private‐voluntary standard labels.^[^ ^8^ ^]^ However, private‐voluntary carbon labels for consumers with higher ecological values, significantly affected their willingness to purchase low carbon products when acceptability, credibility, and understanding of the labels was relatively high.^[^ ^31^ ^]^ Onozaka et al. study on private‐voluntary eco‐labels on locally grown fresh apples for example, found labels with both a where‐grown designation and information on the amount of greenhouse gases emitted from the production and distribution of the product, shifted both supply and demand toward local apple production—reducing carbon emissions 7% during the local production season.^[^ ^9^ ^]^ Similar, Hagman et al. found social norms marketing interventions correct for pluralistic ignorance in sustained ways on campus alcohol use perceptions where most college students overestimate the amount of alcohol their fellow students consume (social norms marketing interventions ostensibly reduce peer‐group pressure to drink).^[^ ^20^ ^]^

### 2.7 Experiential Climate Change Communication

Both studies illuminate how first‐hand learning via experiential communication can leverage the trustworthiness of the information source to educate the public.^[^ ^41^ ^]^ Forced consumer exposure^[^ ^40^ ^]^ to warming labels could be informal sources of climate science education, linking general audiences directly to these harms at the time and place of use, and are an experience‐based opportunity to teach that could increase risk perception attitudes or a belief one is vulnerable to impacts from climate change.^[^ ^35^, ^41^ ^]^

Warming labels as physically and psychologically close experiences of climate change^[^ ^42^ ^]^ are first‐hand learning how immediate harms are generated through one's own actions that may produce self‐efficacy beliefs. Under these conditions, warming labels as domain‐specific, science communication^[^ ^38^ ^]^ could have a powerful unsolicited effect as they play a complementary role in reporting “everyday” acceptable opinion.^[^ ^30^, ^34^ ^]^ Experiential processing is more powerful than analytical processing in driving decision‐making and behavior^[^ ^42^ ^]^ and stimulating interpersonal discussions.^[^ ^37^ ^]^ Therefore, warming labels could have a powerful influence on individual decisions about risk associated with increased levels of saliency and risk perception attitudes.^[^ ^28^, ^30^, ^35^ ^]^

### 2.8 Negative Warming Labels may Effectively Activate Social Norms

Experiential learning at the pump about previously unknown climate‐public health harms is similar to learning about how local weather is affected by man‐made climate change. Zhao et al. found respondents who learn about these effects first‐hand through local TV meteorologists were more certain climate change was happening, understood climate change is primarily human‐caused, and perceived greater harm from climate change.^[^ ^41^ ^]^ Further, their evaluation supported the effectiveness of experiential learning about climate science. Exposure to informal, experienced‐based climate change education—helping viewers understand the causes, processes, and impacts of climate change—reduced the subject from something remote, complex, and confusing to something proximate, concrete, and trustworthy.^[^ ^41^ ^]^

With health warning labels (HWLs), negative messaging may be more effective, possibly due to fear‐based messages producing adaptive, desired responses when both a perceived threat (negative perceptions of graphic HWLs on cigarettes) is high and efficacy (how much someone perceives they would benefit from health and other gains if they quit smoking) is high.^[^ ^11^ ^]^

A 2012 longitudinal study in Canada and Australia found graphic imagery HWLs on 75% of the front of cigarette packs promoted cessation behavior; this was predicted by a stronger threat response to graphic HWLs producing stronger self‐efficacy belief (i.e., individual perceived benefit from quitting).^[^ ^11^ ^]^ However, a review of text‐only alcohol HWLs found them to be ineffective. This may be due to tobacco warning labels implemented in the context of intense and persistent public health campaigns against tobacco smoking where the cultural position and politics of tobacco and alcohol differ.^[^ ^3^ ^]^ On the other hand, an experimental laboratory study where participants viewed HWLs (moderately severe and highly severe) on large beer cans found highly severe HWLs increased an individual's self‐efficacy beliefs; perceiving them as more effective and increasing motivation to drink less.^[^ ^43^ ^]^

Strong self‐efficacy beliefs to quit smoking or drinking may be linked to climate concern norms where warming labels could conjure a high threat response. Too much or too little arousal in such interventions can hinder the climate action performance. At the same time, climate change beliefs and expectations about future conditions are shaped by the physical and social world.^[^ ^1^ ^]^ The greater self‐efficacy to quit smoking for instance, is independently associated with stronger threat responses to graphic HWLs on cigarette packs, but not with rejection of their strong emotional messaging.^[^ ^11^ ^]^

The obvious differences in efficacy between highly severe graphic HWLs and text‐only messages notwithstanding, the (above) results suggest an optimal level of negativity in warming label messaging interacting with an already socially salient climate concern. And perhaps the need for multi‐media campaigns that support warming label programs. Thus, an experiential processing of combustion harms in the case of the Cambridge text, where climate concern is grounded in the social environment, may promote acceptable opinion and activate social norms (we acknowledge the text‐only Cambridge case, versus graphic messaging in HWLs already noted, may yield different efficacy data).

### 2.9 Reducing Emissions is “Normal”

As interdictions, warming labels could generate new discourse about “risky” gasoline consumption, indicating that more aggressive, mitigating actions are needed. Warming labels creating this meta norm can shape new social ideas and arrangements and new emotional states.^[^ ^10^ ^]^ They could foster the belief people have violated a social custom or a legal regulation that becomes a mutually agreed upon social arrangement creating coercion and producing responsibility, with an “audience effect” enhancing the salience of the new norm.^[^ ^10^, ^40^, ^44^ ^]^ This shift would have repercussions on the individual, interpersonal, community, and public policy level^[^ ^35^ ^]^ where it is easier to express one's opinion, and compelling compliance of attitudes and behavior.^[^ ^33^ ^]^

### 2.10 Potential Limitations of Warming Labels

Warming labels of inevitably uncertain environmental and public health outcomes from climate change, can undermine action rather than motivate it.^[^ ^1^ ^]^ As a common “catastrophe narrative”^[^ ^1^ ^]^ the Cambridge text as negative messaging contemporaneously “strands” consumers with their current vehicle and cannot offer an immediate sense of satisfaction one has made a good environmental choice.^[^ ^31^ ^]^

Brewer and Ley found possible obstacles to public standard warming label efficacy where few respondents trust government but exhibited high levels of trust in individual scientists and science media (e.g., science magazines, websites, and TV).^[^ ^26^ ^]^ However, the Cambridge public standard scheme may motivate greenhouse gas reductions through their less ambiguous, educational role grounded in public interest. Government science educating the public on combustion harms and in the absence of more aggressive government action,^[^ ^7^ ^]^ may be a more trusted public source of climate science information (unlike private‐voluntary carbon labels as already noted).

## 3 Discussion

Labels may address the climate policy challenge that “waiting” for the best policy in the near term increases the likelihood of severe impacts.^[^ ^7^ ^]^ Social norms marketing campaigns can shape social ideas, attitudes, and people's values^[^ ^45^ ^]^ in the immediate term. Warming labels may facilitate a collapse in trust in the current (fossil) order, moving systems from one stable state to another.^[^ ^46^ ^]^ Their effectiveness as market‐oriented instruments in motivating reductions in GHG emissions, will depend upon the degree to which they are understood, accepted, and credible to their audience to influence purchasing behavior and willingness to buy into a low carbon consumption context.^[^ ^31^ ^]^ Such schemes could include green education public service announcements and empirical pretesting that considers their “natural setting” to ensure consumers notice and interpret them correctly.^[^ ^7^, ^8^, ^31^ ^]^

Fear, guilt, and shame have been shown to be differently motivating, and the method of coping isn't always problem‐oriented and can result in anger, retreat, and despondency.^[^ ^12^ ^]^ Consumer appraisal of the immediately unresolvable Cambridge text could increase the odds of rejection, perceiving the new government labels as a threat, and the effect of threats on attitude change can “boomerang,” producing defiance rather than compliance.^[^ ^12^, ^47^ ^]^ Fear can motivate people to act if there are clear prescriptions and recommendations on how to act to reduce the threat of climate change.^[^ ^1^ ^]^

Similarly, the Cambridge text may make individuals feel their free behaviors are being threatened^[^ ^48^ ^]^ as they can't wholly resolve the requisite problem shown in the message.^[^ ^12^ ^]^ That said, warming labels exist in the context of other forces—the lack of low‐carbon transportation alternatives for instance—that can motivate a person to give up their fossil fuel privileges and comply with the threat of their elimination.^[^ ^48^ ^]^

Additionally, warming labels targeting conservative audiences, may be rejected. Demographic divides—personal values, education, and income—are factors on people's low carbon consumption behavior and trust in scientific information.^[^ ^26^, ^31^ ^]^ Brewer and Ley point to warming labels as being less salient in rural‐conservative areas where trust in government and science is low and majorities are unconcerned about climate change.^[^ ^26^ ^]^ Labels targeting such subgroup areas may not be corrective of pluralistic ignorance, are likely to reduce discussion among those concerned about climate change, and thus may be unbelievable or disconfirmed to their audience.^[^ ^18^ ^]^

Still, Brewer and Ley found conservatives express less trust in scientists than liberals, but this value was not significantly related to trust in government science.^[^ ^26^ ^]^ Simultaneously, Republican majorities support government policies aimed at reducing the effects of climate change.^[^ ^16^ ^]^ Brewer and Ley results, while more reflective of general not conservative audiences, show high trust in university scientists can “spill over” to government agencies who draw on their credibility, with relatively high trust in individual scientists when respondents didn't connect scientific information to the government.^[^ ^26^ ^]^ Therefore, despite strong rationales and support in the prevailing literature for expecting citizens to evaluate messages about environmental science on the basis of perceived communicator credibility, it would be more useful to determine the extent to which and conditions under which, members of the public do so.^[^ ^26^ ^]^

Notwithstanding, we suggest warming label effectiveness may initially be most appropriate for urban areas with existing climate action planning. For instance, Gately et al. found urban areas responsible for 80% of on‐road emissions growth since 1980 are also emerging hubs of climate change mitigation activity, while continuing to produce the fastest growth in transport‐related CO_2_ emissions.^[^ ^49^ ^]^ Warming label programs would target cities with heavy concentrations of on‐road emissions, may activate existing normative climate concerns^[^ ^18^ ^]^ in these regions.

## 4 Conclusion

By activating climate concern norms, we suggest the Cambridge program as clear, public interest educational messages that do not promote narrow private industry interest, may be trusted and therefore effective in increasing self‐efficacy beliefs.

Vandenbergh et al. suggest label shortcomings don't obviate the value of such programs by providing better information than the consumer has at present.^[^ ^7^ ^]^ This may be especially true in judging the efficacy of warming label programs that may be measured by how “ordinary citizens” engage in climate action^[^ ^1^ ^]^ and build the necessary, long‐term political support for furthering mitigating policies more aggressively.^[^ ^44^ ^]^ Label's ability to educate the public about the urgency of abiding climate‐health effects from combustion could broadly frame and outline an agenda for sustainable transport where social norms have a significant effect on the intention toward green consumerism.^[^ ^31^ ^]^

Labels as present‐day focal points of climate and public health harms could push for technological innovation that leads to a competitive and social advantage.^[^ ^1^ ^]^ Emphasizing individual scientist claims, for example, “Scientists say…,” warming labels may be the most trusted in producing these normative factors. This could make it easier for individuals to achieve difficult individual behavioral changes for good, while producing co‐benefits of climate policies potentially including well‐being and public health benefits from air quality improvements.^[^ ^1^ ^]^

Warming labels can evoke more visceral reactions^[^ ^30^ ^]^ where climate change policy is viewed as too complex and distant from the everyday lives of most people, thus increasing interpersonal talk with corresponding increases in self‐efficacy beliefs associated with long term support for emissions reductions.^[^ ^29^ ^]^ Labels can associate climate action with social values, where climate change includes a moral liability, since it is linked to human suffering.^[^ ^1^ ^]^

Future research questions include how to mobilize largely apathetic audiences to prefer more sustainable transportation options and behaviors. A research hypothesis may state warming labels activating extant climate concern can shape public opinion toward support for more sustainable transport policies and practices. This research could explore how exposure to warming labels may increase the propensity of individuals to undertake adaptive planning (i.e., increase the feeling of self‐efficacy); increase realized uptake of, or intentions for, low carbon transport modes; and shape political participation or voting preferences toward support for low carbon transport.

Regardless, the more linkages made in a way that engages experiential processing of climate effects, and minimizes psychological distance, the more meaningful and effective any such schemes are likely to be.^[^ ^42^ ^]^ We look forward to studies linking exposure to warming labels to realized attitudinal and/or behavioral outcomes as advancing the state of the field.

## 5 Experimental Section

A systematic review was conducted from 1971 through 2021 of the growing theoretical and empirical literature that investigates the relationship between social norms and climate action, based on the approach in Howe et al.^[^ ^42^ ^]^ This literature was reviewed to identify consistencies and inconsistencies, outlining some of the consistencies where warming labels may elevate the opinion of “alarmed” standpoints, shape climate concern norms, were experiential climate communication, and may offer an optimal level of negativity.

A systematic search was conducted on Google Scholar using the following key words: “public opinion,” “social norms marketing,” “climate opinion,” “carbon labels,” and “pluralistic ignorance.” The cited references in the included literature were also searched to identify possible additional publications for inclusion.

The initial search query identified 1490 results. The search was next confined to the first thirteen pages of search results because results were sorted in descending order of search relevance. Inclusion criteria were that:

- The article must be published in a peer reviewed journal.
- Theoretical articles must describe climate opinion, public opinion, social norms, how media shape public opinion, carbon labeling, and climate change communication. Relevant articles not cited within the last five years were excluded.
- Empirical articles must describe or examine at least one construct related to climate opinion, efficacy of carbon labeling programs, efficacy of HWLs, or how social norms marketing could correct for pluralistic ignorance: tendency of carbon label interventions (particular to environmental messaging), social norms marketing programs, HWLs, and experiential learning about climate to alter preferences toward mitigation.

Based on these criteria, 25 empirical articles and 23 theoretical were selected for inclusion. Due to a lack of efficacy data specific to warming labels, building the theoretical case to develop a scaffold for future empirical research that tests the conceptual ideas was emphasized.

## Conflict of Interest

J.R.B. is the founder of the non‐profit Think Beyond the Pump which researches consumer public health and climate warning labels on all points of fossil purchase.
